# Syllogisms delivered in an angry voice lead to improved performance and engagement of a different neural system compared to neutral voice

**DOI:** 10.3389/fnhum.2015.00273

**Published:** 2015-05-12

**Authors:** Kathleen W. Smith, Laura-Lee Balkwill, Oshin Vartanian, Vinod Goel

**Affiliations:** ^1^Department of Psychology, Faculty of Health, York UniversityToronto, ON, Canada; ^2^Humanist Canada, Ottawa, ONCanada; ^3^Department of Psychology, University of Toronto at ScarboroughToronto, ON, Canada; ^4^IRCCS Fondazione Ospedale San CamilloVenice, Italy

**Keywords:** reasoning, emotion, fMRI, anger, sadness, auditory

## Abstract

Despite the fact that most real-world reasoning occurs in some emotional context, very little is known about the underlying behavioral and neural implications of such context. To further understand the role of emotional context in logical reasoning we scanned 15 participants with fMRI while they engaged in logical reasoning about neutral syllogisms presented through the auditory channel in a sad, angry, or neutral tone of voice. Exposure to angry voice led to improved reasoning performance compared to exposure to sad and neutral voice. A likely explanation for this effect is that exposure to expressions of anger increases selective attention toward the relevant features of target stimuli, in this case the reasoning task. Supporting this interpretation, reasoning in the context of angry voice was accompanied by activation in the superior frontal gyrus—a region known to be associated with selective attention. Our findings contribute to a greater understanding of the neural processes that underlie reasoning in an emotional context by demonstrating that two emotional contexts, despite being of the same (negative) valence, have different effects on reasoning.

## Introduction

It has been demonstrated that whereas reasoning with neutral material was associated with activation in left dorsolateral prefrontal cortex, reasoning with negatively charged (provocative) emotional material was associated with activation in ventromedial prefrontal cortex; furthermore, these neural mechanisms were activated in a reciprocal manner (Goel and Dolan, 2003b). Smith et al. (2014) found that, when emotion was induced by positively or negatively valenced pictorial stimuli prior to the introduction of the reasoning task, reasoning about neutral material led to dissociable neural patterns depending on whether the induction had been positive, negative, or neutral. For example, direct comparison of neural activation in the reasoning time windows in the positive and negative conditions, after controlling for baseline effects, yielded activation in cerebellar vermis and right inferior frontal gyrus (orbitalis) after positive emotion induction but activation in left caudate nucleus and left inferior frontal gyrus (opercularis) after negative emotion induction.

In the current study, we continue our investigation of the effect that emotion has on reasoning. Whereas the previous studies examined the effects of visually presented emotional syllogism content, and visually presented emotional valence (positive and negative), here our interest is to discover whether reasoning and its neural underpinnings will be affected differently by exposure to the expression of two different emotions in the auditory channel.

There is support from various theoretical models in the literature for the existence of different specific emotions, each with its own neural and/or physiological signature (Friedman, 2010); moreover, individuals in therapy can be guided to switch from one specific emotion to another by methods designed to alter their underlying physiology and therefore their current emotional experience (Smith and Greenberg, 2007). Appraisal models likewise consider the differential effects of specific emotions such as dispositional fear and anger on the evaluation of subsequently occurring events (Lerner and Keltner, 2001; DeSteno et al., 2004; Dunn and Schweitzer, 2005).

Our interest in testing the effects of specific emotions (rather than emotional valence) is that we hope to show that reasoning and its neural underpinnings are affected differently by expression of different specific emotions. We chose anger and sadness as the specific emotions because there is literature (to be presented next) suggesting that these emotions are characterized differently.

The neuroimaging literature provides evidence that sadness and anger are characterized differently. A meta-analysis of neuroimaging of emotion (Murphy et al., 2003) reported that whereas anger has been associated with the lateral orbitofrontal cortex, happiness and sadness have been associated with supracallosal anterior cingulate and dorsomedial prefrontal cortex.

Neural activation associated with hearing the voice of an angry speaker (Sander et al., 2005) was noted in bilateral superior temporal sulcus (right BA 42, bilateral BA 22) and right amygdala. Grandjean et al. (2005) demonstrated that superior temporal lobe activation associated with anger prosody is associated with the angry emotion itself, and not with low-level acoustical properties of the stimulus. Other activations found by Sander et al. (2005) include cuneus, left superior frontal gyrus (BA 8), right medial orbitofrontal cortex, left lateral frontal pole (BA 10), right superior temporal sulcus (BA 39), and bilateral ventrolateral prefrontal cortex (BA 47). Ethofer et al. (2009) investigated whether neural activation to angry versus neutral prosody would depend on the relevance of the prosody to the task; tasks were to judge the affective prosody (angry, neutral) or word class (adjective, noun) of semantically neutral spoken words. Neural activation associated with angry versus neutral prosody was reported in bilateral superior temporal gyrus, bilateral inferior frontal/orbitofrontal cortex, bilateral insula, mediodorsal thalamus, and bilateral amygdala, regardless of task, suggesting that these activations occur automatically when processing emotional information in the voice. Neural activation was greater during judgment of emotion than word classification in bilateral inferior frontal/orbitofrontal cortex, right dorsomedial prefrontal cortex, and right posterior middle and superior temporal cortex. Quadflieg et al. (2008) found that neural activation associated with angry versus neutral prosody was noted in fronto-temporal regions, amygdala, insula, and striatum. Identification of the prosody as emotional was additionally associated with activation in orbitofrontal cortex. Individuals with social phobia, compared to healthy controls, demonstrated a larger response in orbitofrontal cortex in response to angry prosody, regardless of whether the task related to the prosody (identify prosody as emotional or neutral) or not (identify the gender of the speaker).

Neural correlates of sadness invoked by re-experiencing of sad autobiographical episodes (Liotti et al., 2000) were reported in the subgenual anterior cingulate (BA 24/25), right posterior insula, and left anterior insula. Relative deactivation was noted in right dorsolateral prefrontal cortex (BA 9), bilateral inferior temporal gyrus (left BA 20, right BA 20/37), right posterior cingulate/retrosplenial cortex, and bilateral parietal lobes.

A second reason for choosing anger and sadness is that these emotions have been posited to have different effects on attention, memory, and categorization (Gable and Harmon-Jones, 2010b) and therefore may have different effects on reasoning.

In theoretical terms, anger is an important emotion because despite its negative valence it is an ‘approach-related’ emotion, and this observation has prompted a reconsideration of theoretical models of emotion (Carver and Harmon-Jones, 2009). Carver and Harmon-Jones (2009) proposed a model incorporating discrete emotions such as joy, anger, calm, and fear into a dimensional model combining approach/withdrawal with system functioning (i.e., events going well or poorly). In this model, anger is classified as an approach emotion activated when system functioning is going poorly.

Following on this, Gable and Harmon-Jones (2010b) proposed a model outlining the consequences for attention, memory, and categorization of emotions classified on the dimensions of approach/withdrawal in relation to an object or goal, coupled with the strength of that motivation. Specifically, disgust and fear may be strong motivators to avoid an object or goal whereas sadness may be a mild motivator to withdraw from an object or goal. Anger, in contrast, may be a strong motivator to approach an object or goal, despite being negative in valence (Carver and Harmon-Jones, 2009). Regarding the consequences of a strong motivator (such as anger) and a weak motivator (such as sadness) on attention, converging evidence (see Gable and Harmon-Jones, 2010b for a review) suggests that strong motivation to either approach or avoid an object or goal is associated with narrowed attention toward that object or goal, and a lack of attention to other stimuli in the environment that are not relevant to that goal. In contrast, weak motivation, which may occur post-goal-attainment, is associated with broadened attention toward more information from the environment beyond the goal itself.

Consistent with the Gable and Harmon-Jones (2010b) model, lab-induced anger and fear have (separately) led to selective attention to targets at the expense of non-target information (Finucane, 2011); so has disgust (Gable and Harmon-Jones, 2010a). Brosch et al. (2008) reported that angry prosody facilitated selective attention to a concurrently presented visual stimulus.

In contrast, sadness has led to a broadening of attention to global rather than local features of stimuli (Gable and Harmon-Jones, 2010a).

As has been noted above, anger is often studied using an auditory paradigm. Accordingly, we decided to use an auditory paradigm in the current study. Auditory paradigms have been used previously to study reasoning in the absence of emotion (Knauff et al., 2002, 2003; Fangmeier and Knauff, 2009).

Finally, we chose to deliver the reasoning material concurrently with the emotive (and neutral) tones of voice, rather than subsequent to the different tones of voice. Our choice was pragmatic: the latter design would have resulted in a longer experiment, and therefore longer scanning time.

Therefore, our study investigated whether reasoning about neutral material would be affected if the content were presented in sad, neutral, or angry tone of voice. To address this issue, we constructed a 3 (Emotion) × 2 (Task) within-subjects design, where the three levels of the Emotion factor were sad, neutral, and angry, and the two levels of the Task factor were reasoning and baseline.

In Smith et al. (2014), the negative and positive valence inductions were each comprised of a mix of emotions, and we found that reasoning tended to be impaired after each valence of emotion. In the current study, our choice of two specific negative emotive tones of voice, anger and sadness, was motivated by the expectation that each of these specific expressions of emotion would lead to different reasoning performance and different underlying neural characteristics. Thus, our hypothesis was that the neural systems underlying reasoning (involving syllogisms with neutral content) following exposure to each of angry and sad emotion expression would differ from the neural underpinnings of reasoning in the neutral condition, and would thereby elucidate the mechanisms underlying differences in reasoning performance in the two emotional contexts.

## Materials and Methods

### Participants

Data were acquired from 17 participants (10 males, 7 females). Education levels ranged from partially completed undergraduate study to completed graduate degrees, with a mean of 16 years (SD = 2.04) of education. Ages ranged from 20 to 38 (mean 26.5 years, SD 5.95).

The study was approved by the York University Research Human Participants Ethics Committee. All participants gave informed consent.

### Stimuli

Reasoning stimuli consisted of 80 syllogisms that were emotionally neutral in content. The arguments in 39 of these syllogisms were logically valid whereas the arguments in the remaining 41 were logically invalid. Examples of syllogisms are “All gentle pets are canines. Some kittens are gentle pets. Some kittens are canines” (which is valid), and “No fruits are fungi. All mushrooms are fungi. Some mushrooms are fruits” (which is invalid).

As well, there were 40 baseline “syllogisms,” in which the concluding sentence was taken from a different syllogism in the dataset, thereby ensuring that the conclusion of the baseline would be unrelated to the content of the two premises. An example of a baseline trial is “Some movie-goers are men. All men are French. No people are priests.” Thus, the baseline trials provide a control for the reasoning trials, in that the following processes are held constant across both types of trials: hearing the speaker deliver sentences with neutral semantics, hearing the emotion in the tone of voice (constant within each condition), learning the two premises of each argument, and preparing to engage in reasoning. Crucially, what is *not* held constant is that, in a baseline trial, the participant would disengage from the reasoning process instead of making any attempt to integrate the “conclusion” into the premises.

We controlled for the effect of belief-bias (Evans, 2003; Goel and Dolan, 2003a) by ensuring the reasoning syllogisms were balanced overall for validity and for congruence between logic and beliefs. Congruence occurs when the argument logic is valid and the conclusion is believable or when the argument logic is invalid and the conclusion is unbelievable. Incongruence occurs when the argument logic is valid and the conclusion is unbelievable or when the argument logic is invalid and the conclusion is believable.

Congruent syllogisms, incongruent syllogisms, and baselines were chosen (during study design) for each level of the Emotion factor (Sad, Neutral, and Angry). Then the order of the 120 trials was randomized. Finally, the trials were segregated into three presentation sets of 40 trials each. The order of presentation of these three sets was counterbalanced among participants, one set for each session (“run”) in the scanner.

All stimuli had been pre-recorded by the same female speaker (Laura-Lee Balkwill). Among the 80 reasoning syllogisms, the tone of voice was sad for 20, angry for 20, and neutral for 40 stimuli. Among the 40 baseline “syllogisms,” the tone of voice was sad for 10, angry for 10, and neutral for 20 stimuli. Please refer to the Supplementary Material for a discussion concerning the frequency of baseline trials. The intended expression of emotion of all of the stimuli was determined by a separate pilot test involving 15 participants who did not participate in the main experiment. See Appendix A for details.

### Study Design

**FIGURE 1:**
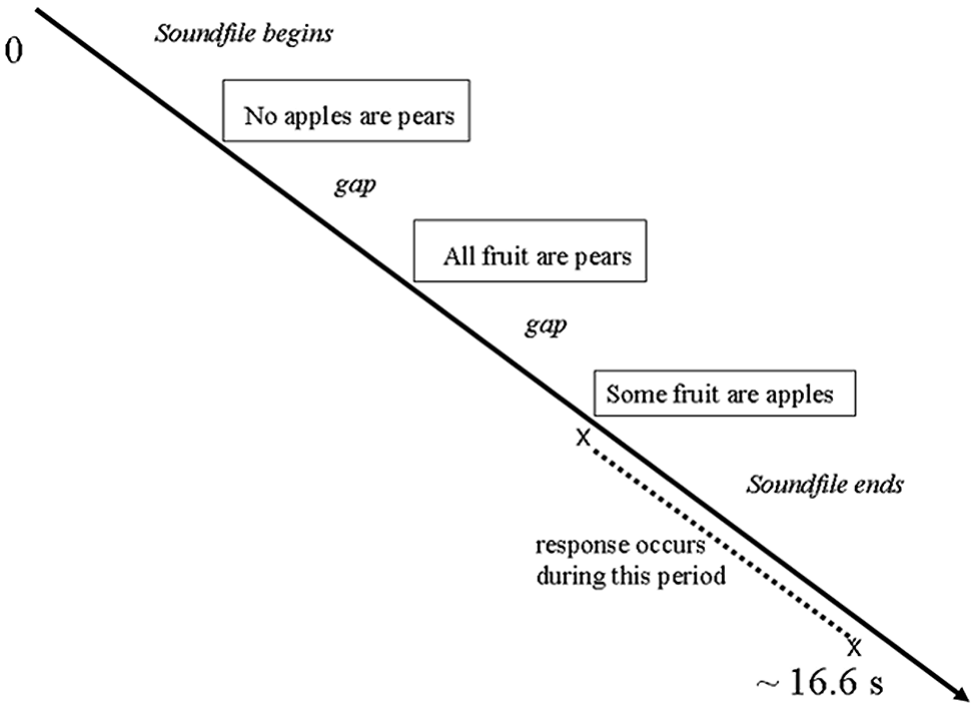
**Design of each trial**.

Each trial involved the following presentation sequence (see **Figure 1**): On each trial, the participant listened to a syllogism through earphones; the task was to press one of two keys to indicate whether or not the conclusion followed logically from the two previous statements. Each participant used one hand for both responses; choice of hand was counterbalanced among participants. Soundfiles varied in length from 7.4 to 15.6 s (mean 10.74 s, SD 1.77 s). However, presentation of the next sound stimulus was not entrained to the preceding response but was timed to be in synchrony with the acquisition of the brain scans. Therefore, trials varied in length from 16.53 to 16.74 s (mean 16.65 s, SD 0.024 s).

### *f*MRI Scanning Technique

A 1.5T Siemens VISION system (Siemens, Erlangen, Germany) was used to acquire T1 anatomical volume images (1 mm × 1 mm × 1.5 mm voxels) and T2^∗^-weighted images (64 × 64, 3 × 3-mm pixels, TE = 40 ms), obtained with a gradient echo-planar sequence using blood oxygenation level-dependent (BOLD) contrast. Echo-planar images (2-mm thick) were acquired axially every 3 mm, positioned to cover the whole brain. Each volume (scanning of the entire brain) was partitioned into 36 slices, obtained at 90 ms per slice. Data were recorded during a single acquisition period. Volume (vol) images, 215 volumes per session, were acquired continuously, for a total of 645 volume images over three sessions, with a repetition time (TR) of 3.24 s/vol. The first six volumes in each session were discarded (leaving 209 volumes per session) to allow for T1 equilibration effects.

### Data Analysis

#### Behavior

Behavioral data were analyzed using SPSS, version 16.0 (SPSS Inc., Chicago, IL, USA).

Note that we shall refer to the conditions as ‘anger,’ ‘sad,’ and ‘neutral,’ for ease of reading, rather than repeating ‘expression of.’

Data from 15 of the original 17 participants were usable in the neuroimaging analysis (data from two participants were discarded because of head movement greater than 2 mm during scanning); therefore, the behavioral analyses are based on 15 participants. As well, one person’s data for the third run (session) were discarded because of lack of engagement in the task. There were a total of 1760 trials remaining: 1175 reasoning (66.76%) and 585 baselines (33.24%). Fifty percentage of trials were neutral; 25% were sad, and 25% were angry. Thus, half of all trials were neutral and half were emotional.

#### Neuroimaging

The functional imaging data were preprocessed and subsequently analyzed using Statistical Parametric Mapping SPM8 (Friston et al., 1994; Wellcome Department of Imaging Neuroscience^1^).

All functional volumes were spatially realigned to the first volume. All volumes were temporally realigned to the AC–PC slice, to account for different sampling times of different slices. A mean image created from the realigned volumes was coregistered with the structural T1 volume and the structural volumes spatially normalized to the Montreal Neurological Institute brain template (Evans et al., 1993) using non-linear basis functions (Ashburner and Friston, 1999). The derived spatial transformation was then applied to the realigned T2^∗^ volumes, which were finally spatially smoothed with a 12 mm FWHM isotropic Gaussian kernel in order to make comparisons across subjects and to permit application of random field theory for corrected statistical inference (Worsley and Friston, 1995). The resulting time series across each voxel were high-pass filtered with a cut-off of 128 s, using cosine functions to remove section-specific low frequency drifts in the BOLD signal. Global means were normalized by proportional scaling to a grand mean of 100, and the time series temporally smoothed with a canonical hemodynamic response function to swamp small temporal autocorrelations with a known filter.

During each trial, the participant listened to the aural delivery of premise one, premise two, and the conclusion of the syllogism. This was followed by a period of silence during which the participant could indicate, by a keypress, whether or not the conclusion logically followed from the first two statements. During neuroimaging data analysis, the emotion expression time window was defined as “listening to premise one and premise two, plus the gap following premise two.” The reasoning time window was defined as “the gap from offset of the conclusion up to but not including the actual motor response.” Each of these time windows was analyzed separately.

Within each stimulus soundfile, the mean decibel level was calculated for the time segment corresponding to each brain scan that had been acquired. During the first level of neuroimaging analysis, described below, the potential confound of mean decibel level was covaried out.

Condition effects at each voxel were estimated according to the general linear model and regionally specific effects compared using linear contrasts. Each contrast produced a statistical parametric map of the *t*-statistic for each voxel, which was subsequently transformed to a unit normal *Z*-distribution. The BOLD signal was modeled as a canonical hemodynamic response function with time derivative.

##### Emotion Expression Time Window

All events from the emotion expression time window (sad, angry, and neutral listening) were modeled in the design matrix as epochs, and events of no interest (conclusion, thinking, and motor response) were modeled out. Sad, angry, and neutral listening were each modeled as an epoch from onset of premise one, with duration being the length of the syllogism *minus* the length of the conclusion. Onset for the conclusion condition was the start of hearing the conclusion; onset for the thinking condition was the end of hearing the conclusion; and onset for the motor response was the scan being acquired at the onset time of each motor response for each participant for each trial. Mean decibel level for each scan was covaried out during this first level analysis.

Contrast images were subsequently analyzed at the group level. A one-way univariate analysis of variance (ANOVA), within-subjects, was conducted with three conditions of interest (sad, angry, and neutral) and 15 subject conditions, with correction for non-sphericity. The analysis generates one *F* test for the effects of interest. The *F* test generated a statistical parametric map of the *F-ratio* for each voxel. The subsequent comparisons each generated a statistical parametric map of the *t*-statistic for each voxel, which was subsequently transformed to a unit normal *Z*-distribution. The activations reported in Supplementary Table S1 survived a threshold of *p* < 0.005 using a random effect model and an extent of 180 voxels. This choice of threshold and extent corresponds to a corrected *p* < 0.05 using the AlphaSim program^2^ with parameters (FWHMx = 8.35 mm, FWHMy = 6.59 mm, FWHMz = 7.74 mm, within the avg152T2.nii mask from the SPM toolbox). The real smoothness in the three directions was estimated from the residuals by using 3dFWHMx. (This AlphaSim procedure was also used during the reasoning time-window, with the following parameters: FWHMx = 8.33 mm, FWHMy = 6.58 mm, FWHMz = 7.71 mm.)

##### Reasoning Time Window

For first-level analysis of the reasoning window, the scans acquired while the participant was engaged in reasoning were modeled as epochs by task (reasoning, baseline) and emotion (sad, angry, neutral) whereas all other conditions (Premise 1, Premise 2, Conclusion, motor response) were modeled out as events of no interest.

Onset for the six Emotion × Task conditions was the end of the conclusion sentence. Duration was from that moment until the individual participants’ motor response within each trial. However, for those trials where there was no response, or the response occurred after the start of the next trial, the duration was set as “start of the next soundfile *minus* 200 ms.” For those trials where participants responded during the concluding sentence (6% of trials), the duration was set as 100/3240 (that is, 0.03 TR); this strategy allowed us to include the contrast image (rather than having an unbalanced design) while ensuring minimal contribution of the activations to the analysis. Onset for each premise and the conclusion was the beginning of the relevant sentence; onset of the motor response was the millisecond at which that response occurred. Thus, altogether, 10 (conditions) × 3 (sessions) contrast images were generated for each participant. Mean decibel level for each scan was covaried out.

Contrast images were subsequently analyzed at the group level. A one-way univariate ANOVA was conducted, within-subjects, with six conditions of interest (sad reasoning, sad baseline, angry reasoning, angry baseline, neutral reasoning, neutral baseline) and 15 subject conditions, with correction for non-sphericity. The analysis generates one *F* test for the effects of interest.

The *F* test and the subsequent *a priori* comparisons each generated a statistical parametric map of the *t*-statistic for each voxel, which was subsequently transformed to a unit normal *Z*-distribution. The activations reported in Supplementary Table S2 survived a threshold of *p* < 0.005 using a random effect model and an extent of 180 voxels. (See the above description regarding the emotion expression time-window for details.)

## Results

### Behavioral Results

The overall percentage of correct responses on the reasoning trials was 66.9%. For baselines (where the correct response would always be “not valid”), the percentage of correct responses was 99.3%. Mean reaction time, after presentation of the third sentence, on reasoning trials was 2211 ms (SD 1121), and on baseline trials it was 472 ms (SD 112). This difference was significant: paired *t*(14) = -6.366, *p* = 0.001.

**FIGURE 2:**
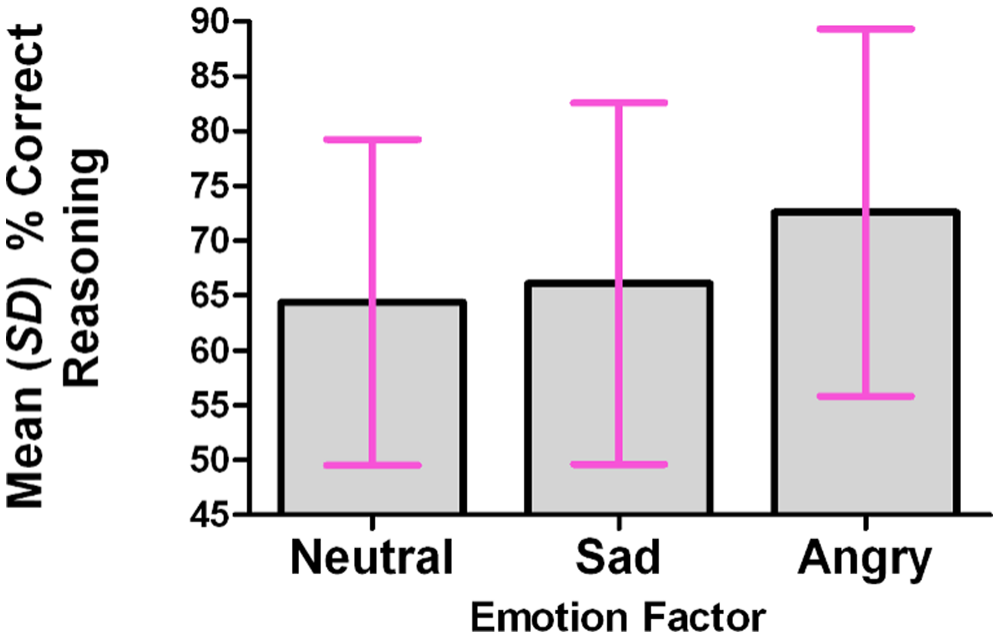
**The percentage of correct reasoning responses was significantly higher in the angry condition than in the neutral or sad conditions**.

For each participant, the percentage of correct responses was calculated within each level of the Emotion factor. A repeated-measures analysis was conducted, using the multivariate approach; the omnibus test was significant: *F*(2,13) = 4.084, *p* = 0.042. The Emotion factor (tone of voice) accounted for 38.6% of the total variance in the percentage of correct responses. The percentage of correct responses was significantly higher in the Angry condition than in the Neutral condition (*p* = 0.031, corrected for multiple comparisons using Bonferroni). See **Figure 2**.

Mean percentages of correct responses were as follows: neutral 64.4% (SD 14.9); sad 66.1% (SD 16.5); angry 72.6% (SD 16.7).

A repeated-measures analysis of response time on correct responses was conducted across the Emotion factor. There was no significant difference among the means (*p* = 0.818). Mean reaction times were as follows: neutral 1599 ms (SD 480); sad 1626 ms (SD 672); angry 1671 ms (SD 573).

### Neuroimaging Results

#### Emotion Expression Time Window

**FIGURE 3:**
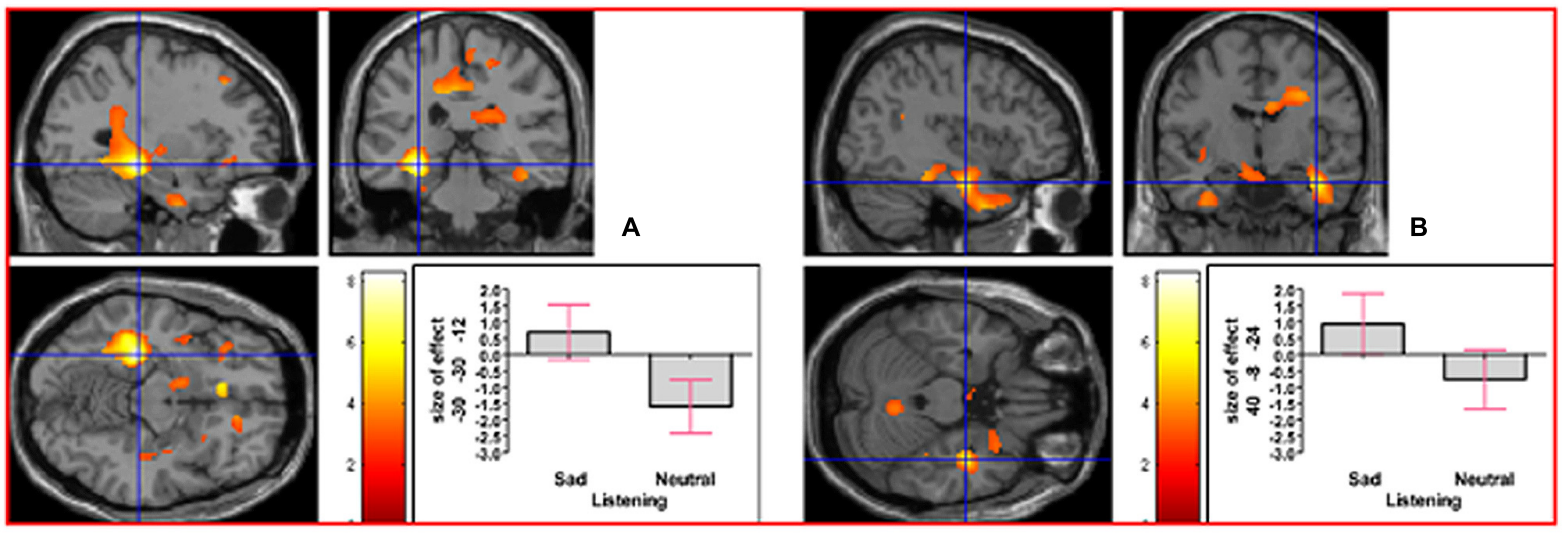
**The contrast (Sad - Neutral) elicited activation in (A) left hippocampus (MNI co-ordinates: -30, -30, -12, cluster size 6766 voxels, *Z* = 5.83), and in (B) right hippocampus (MNI co-ordinates: 40, -8, -24, cluster size 1135 voxels, *Z* = 4.51)**. There was also activation in left inferior temporal gyrus and in right primary somatosensory cortex (not shown).

**FIGURE 4:**
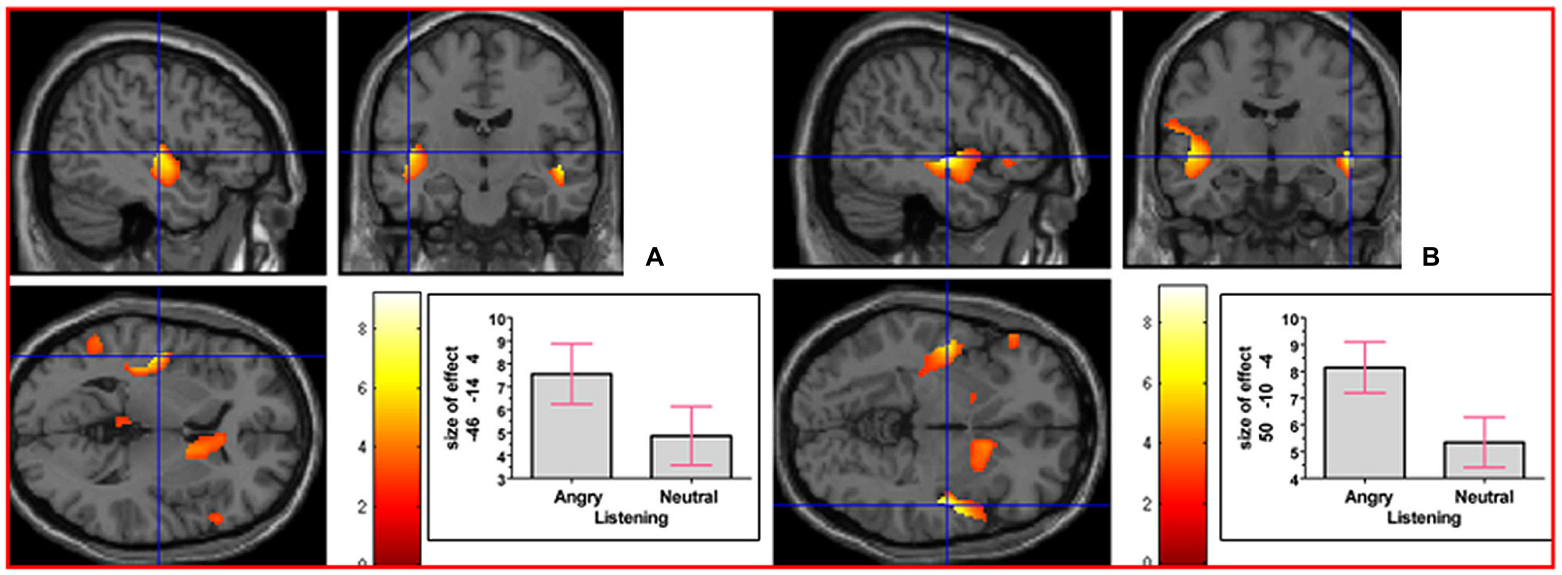
**The contrast (Angry - Neutral) elicited activation in (A) left superior temporal gyrus (MNI co-ordinates: -46, -14, 4, cluster size 746 voxels, *Z* = 5.01), and in (B) right superior temporal gyrus (MNI co-ordinates: 50, -10, -4, cluster size 463 voxels, *Z* = 6.20)**. There was also activation in right supramarginal gyrus (not shown).

As indicated in Supplementary Table S1, in the contrast (Emotion - Neutral), relative deactivation was found in left hippocampus extending into left insula and relative activation was found in right posterior insula extending into right inferior temporal gyrus. The reverse contrast, namely (Neutral - Emotion), yielded relative deactivation in left inferior frontal gyrus (opercularis, extending into triangularis area 45) and in left precentral gyrus extending into left superior frontal gyrus. The contrast (Sad - Neutral, masked inclusively with Emotion - Neutral at *p* = 0.05) yielded relative activation in left hippocampus extending into left precuneus, in right hippocampus extending into right inferior temporal gyrus and right fusiform, in left inferior temporal gyrus extending into left hippocampus and fusiform, and in right primary somatosensory cortex extending into right precentral gyrus (area 6; see **Figure 3**). The reverse contrast (Neutral – Sad) yielded relative activation in left superior temporal gyrus extending into middle temporal gyrus, in right superior temporal gyrus, relative deactivation in left cerebellum extending into right cerebellar vermis, in left inferior frontal gyrus (opercularis: area 44), in left calcarine gyrus (area 17), and in right cerebellum. The contrast (Angry - Neutral, masked inclusively with Emotion - Neutral at *p* = 0.05) yielded relative activation in left superior temporal gyrus, in right superior temporal gyrus, and in right supramarginal gyrus extending into right superior temporal gyrus (see **Figure 4**). The reverse contrast (Neutral - Angry) yielded relative deactivation in left superior frontal gyrus (area 6), in left supramarginal gyrus, and in right angular gyrus. The contrast (Sad - Angry, masked inclusively with Emotion - Neutral at *p* = 0.05) yielded relative activation in left hippocampus extending into left cuneus, and in right hippocampus extending into right inferior temporal gyrus. The reverse contrast (Angry - Sad, masked inclusively with Emotion - Neutral at *p* = 0.05) yielded relative activation in left superior temporal gyrus extending into secondary somatosensory cortex, and in right superior temporal gyrus.

#### Reasoning Time Window

As indicated in Supplementary Table S2, analysis of the main effect of (Reasoning - Baseline) yielded relative activation in right insula extending into right caudate nucleus, in left precentral gyrus extending into left primary somatosensory cortex, and in left insula extending into left inferior frontal gyrus (triangularis). Analysis of the main effect (Emotional Reasoning - Emotional Baseline) yielded relative activation in right thalamus (temporal) extending into right insula, in left precentral gyrus extending into left primary somatosensory cortex, and in right middle cingulate cortex.

For results of simple effect analyses please refer to the Supplementary Material including Supplementary Table S2.

**FIGURE 5:**
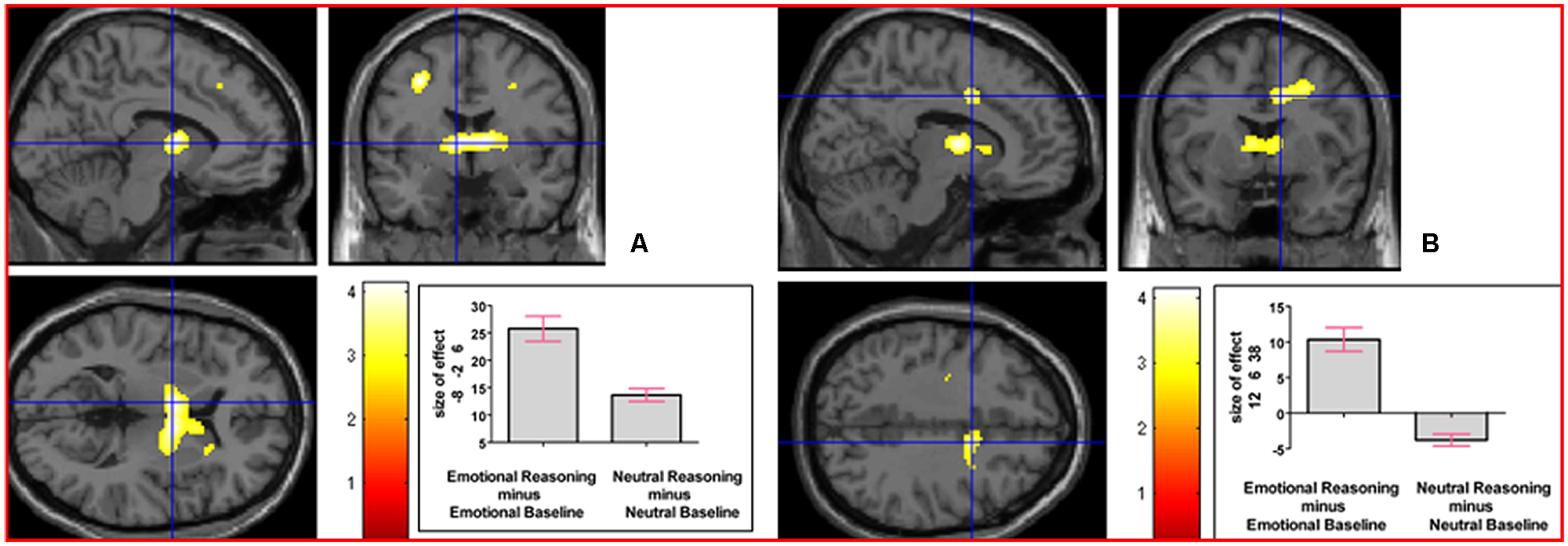
**The interaction contrast [(Emotional Reasoning - Emotional Baseline) - (Neutral Reasoning - Neutral Baseline)] elicited activation in (A) left thalamus (MNI co-ordinates: -8, -2, 6, cluster size 832 voxels, *Z* = 3.88), and in (B) right middle cingulate cortex (MNI co-ordinates: 12, 6, 38, cluster size 311 voxels, *Z* = 3.47)**.

We next addressed the question of whether neural activation underlying reasoning in an emotional context, collapsed across the emotion factor, would differ from that underlying neutral reasoning. The interaction contrast [(Emotional Reasoning - Emotional Baseline) - (Neutral Reasoning - Neutral Baseline)] yielded relative activation in left thalamus (temporal) extending into right thalamus (temporal) and right caudate nucleus, and in right middle cingulate cortex (see **Figure 5**). For details of the reverse interaction contrast, see the Supplementary Material including Supplementary Table S2.

To determine whether neural activation underlying reasoning in the sad and neutral time windows would differ, we analyzed the interaction contrast [(Sad Reasoning - Sad Baseline) - (Neutral Reasoning - Neutral Baseline)]; this analysis yielded no clusters surviving the specified extent. For details of the reverse interaction contrast, see the Supplementary Material including Supplementary Table S2.

**FIGURE 6:**
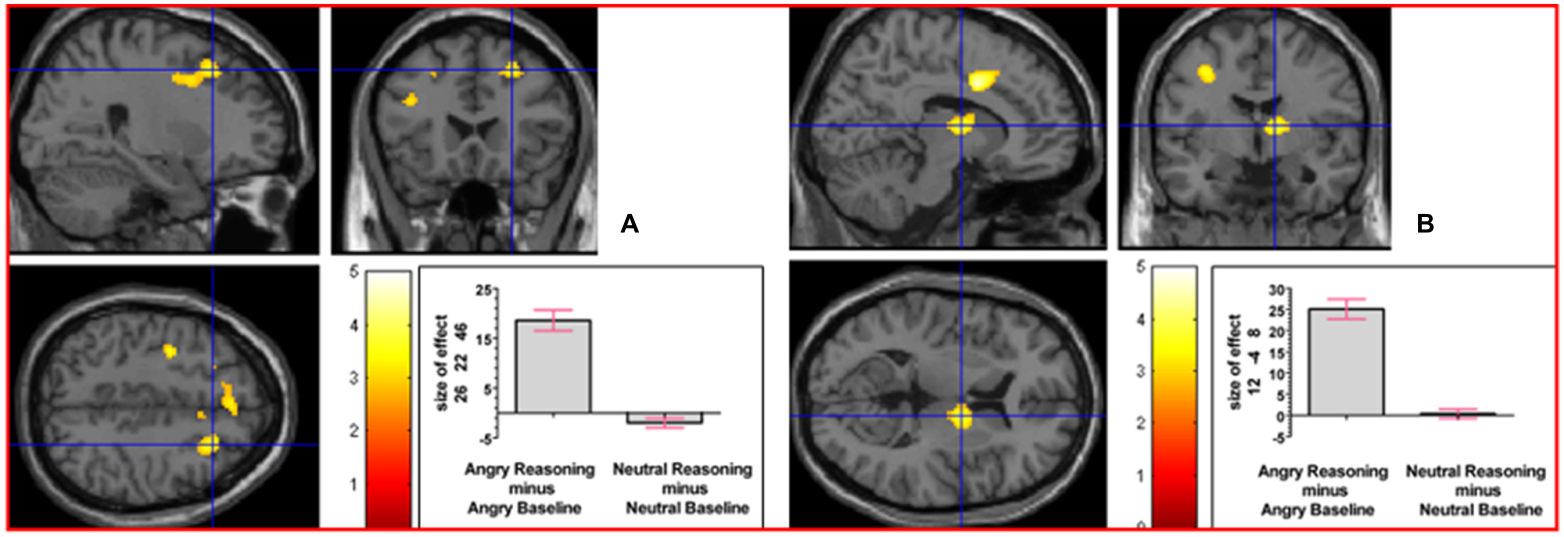
**The interaction contrast [(Angry Reasoning - Angry Baseline) - (Neutral Reasoning - Neutral Baseline)] elicited activation in (A) right superior frontal gyrus (MNI co-ordinates: 26, 22, 46, cluster size 611 voxels, *Z* = 3.43), and in (B) right thalamus (MNI co-ordinates: 12, -4, 8, cluster size 220 voxels, *Z* = 3.67)**.

To determine whether neural activation underlying reasoning in the angry and neutral time windows would differ, we analyzed the interaction contrast [(Angry Reasoning - Angry Baseline) - (Neutral Reasoning - Neutral Baseline)]; this analysis yielded relative activation in right superior frontal gyrus and in right thalamus (prefrontal; see **Figure 6**). For details of the reverse interaction contrast, see the Supplementary Material including Supplementary Table S2.

To determine whether neural activation underlying reasoning in the sad and angry time windows would differ, we analyzed the interaction contrast [(Sad Reasoning - Sad Baseline) - (Angry Reasoning - Angry Baseline)] and also the reverse interaction contrast [(Angry Reasoning - Angry Baseline) - (Sad Reasoning - Sad Baseline)]; neither of these interaction contrasts yielded any clusters surviving the specified extent.

To determine whether there would be any activations in common between sad reasoning and angry reasoning after accounting for their respective baselines, we conducted a conjunction analysis of the two interaction contrasts [(Sad Reasoning - Sad Baseline) - (Neutral Reasoning - Neutral Baseline)] and [(Angry Reasoning - Angry Baseline) - (Neutral Reasoning - Neutral Baseline)]; however, there were no suprathreshold clusters.

## Discussion

### Engagement with the Task

First, we consider whether participants were engaged in the reasoning task, by looking first at the behavioral and then at the neural results. Behaviorally, we note that accuracy levels were above chance. At the neural level, we have reported caudate nucleus involvement in several reasoning contrasts, including the main effect of reasoning. Such findings are consistent with the important role of basal ganglia in the reasoning process, as reported in the literature (Goel et al., 2000; Christoff et al., 2001; Melrose et al., 2007; Smith et al., 2014).

### Success of Tone of Voice Manipulations

Second, we consider whether our tone of voice manipulations were successful. Reasoning performance in the sad condition was neither impaired nor improved compared to reasoning in the neutral condition. However, reasoning performance in the angry condition was better than in the neutral tone of voice condition. If we were to consider only the behavioral results, we might conclude that the sad tone of voice was ineffective. However, the pattern of neural results indicates that each of the two tones of voice were successful: During the listening time window, each emotive tone of voice condition yielded a different pattern of neural activation. Specifically, the contrast “sad *minus* neutral” activated a different neural pattern than did the contrast “anger *minus* neutral.” As well, the contrasts “sad *minus* angry” and “angry *minus* sad” yielded different patterns of neural activation. Thus, evidence shows that while participants were listening to the syllogism, they were being affected, concurrently, by the emotion expression, whether in the sad or in the angry condition.

The field of emotion research still has much to learn about the decoding and interpretation of auditory anger; thus, we should consider the possibility that our ‘anger’ stimuli invoked responses in the participants that would be more associated with fearful expression than expression of anger. We did not obtain emotion ratings during scanning, nor did we acquire peripheral psychophysical measurements from study participants. However, converging evidence from the pilot study of stimuli ratings and from other sources points more toward ‘anger’ than toward ‘fear.’

During the pilot study, participants had the opportunity on 50% of trials to reject both ‘sad’ and ‘angry’ as ratings in favor of writing down a preferred term; nevertheless no participant wrote ‘fear’ for any stimulus. On the other 50% of trials, participants were asked to rate stimuli in terms of being active (goal-oriented) or passive (no goal) rather than choosing an emotion term. Only one participant rated one ‘angry’ stimulus as passive. On 100% of trials, participants indicated how sure they were of each rating; for each of sad and angry, people indicated ‘yes’ or ‘definitely’ (rather than ‘maybe’) on 29 out of 30 stimuli being rated. Please refer to Appendix A for details. Secondly (see below), neural activation associated with anger expression in the current study was similar to that reported by Grandjean et al. (2005). We did not find any neural activation in amygdala, a neural region often associated with fear (LeDoux, 1996; van Well et al., 2012; Adolphs, 2013).

### Interpretation of Findings Regarding Reasoning in an Angry Context

We now consider how the findings regarding reasoning in an angry context should be interpreted. In two separate studies, induced anger has been shown to enhance heuristic rather than analytical processing (Bodenhausen et al., 1994; Tiedens and Linton, 2001). In contrast, Gable and Harmon-Jones (2010b) proposed that emotions such as anger that are associated with high motivation toward a goal should promote selective attention toward a target and away from irrelevant distraction. Indeed, that model fits well with our behavioral findings, which were that reasoning (the target task) improved after angry tone of voice (which was not the focus of the assigned task) compared to reasoning after neutral tone of voice.

As reported above, neural activation associated with hearing the voice of an angry speaker (Sander et al., 2005) was noted in bilateral superior temporal sulcus (right BA 42, bilateral BA 22), and right amygdala; Grandjean et al. (2005) demonstrated that superior temporal lobe activation associated with anger prosody is associated with the angry emotion itself, and not with low-level acoustical properties of the stimulus. Sander et al. (2005) utilized a dichotic listening task, which was to attend to the left- or right-ear presentation and identify the gender of the speaker; there was no instruction associated with the speaker’s angry or neutral tone of voice. The above findings (in Sander et al., 2005) were for angry prosody regardless of whether attended or not; however, neural data were also analyzed separately for the attended and unattended ear of presentation. There was a tendency (in Sander et al., 2005) for activation in orbito-frontal cortex to increase in the attended-side angry prosody condition and to decrease in the unattended-side neutral prosody condition. Also, there was a tendency for activation in bilateral ventro-lateral prefrontal cortex to increase in the attended-side angry prosody condition. There was also activation in right cuneus associated with attended anger, but this activation did not survive correction for multiple comparisons. In the current study, we noted activation in *left* cuneus associated with the angry *reasoning* condition, but we did not find any activations in orbito-frontal cortex, ventro-lateral prefrontal cortex, right cuneus, or amygdala, in either the angry listening time window or the angry reasoning time window. Thus, neural activations previously associated with attention to the anger prosody were not apparent among our findings.

Selective attention has often been associated with neural activation in right superior frontal gyrus (see the review by Corbetta and Shulman, 2002). In the current study, reasoning in the angry condition was found to be associated with significant activation in right superior frontal gyrus and in right thalamus.

Thus, converging behavioral and imaging evidence suggests that, during the listening time window, angry tone of voice led to activation of neural regions previously associated with unattended anger; subsequently, during the (silent) reasoning time window, a neural region previously associated with selective attention toward the main task (in this case, reasoning) was recruited and participants’ level of reasoning performance was sharper than it was after neutral tone of voice.

### Interpretation of Findings Regarding Reasoning in a Sad Context

Clearly, a different mechanism was at work as a result of the expression of sad tone of voice. As we indicated above, the expressed sadness itself was effective, leading to a differentiated pattern of neural activation during the listening time window. Looking at past literature, we note that auditory induction of sadness, using sad classical music, led to activation in hippocampus/amygdala and auditory association areas (Mitterschiffthaler et al., 2007); as in that study, our use of sad expression led to extensive activation in hippocampus during the listening time window. However, in Mitterschiffthaler et al. (2007) participants were directed to pay attention to their emotional experience during scanning. A different study showed that emotional memories, but not neutral memories, have been associated with hippocampal and amygdala activation (Dolcos et al., 2004). Therefore, we propose that in the current study, participants were attending to the sad tone of voice while simultaneously learning the syllogism. However, given that reasoning performance in the sad condition was comparable to that in the neutral condition, we conclude that sad emotive tone of voice did not significantly impact the reasoning process itself.

## Conclusion

We have contributed to a deeper understanding of the characterization of specific emotions, by demonstrating that two contexts of expressed emotion, each being of negative valence, have nevertheless different effects on reasoning. Unlike sad auditory context, logical reasoning in an angry auditory context is characterized by increased accuracy, and is accompanied by recruitment of an underlying neural system known to be associated with selective attention. These results increase our understanding of the neural processes that underlie reasoning in the context of auditory emotion.

## Conflict of Interest Statement

The authors declare that the research was conducted in the absence of any commercial or financial relationships that could be construed as a potential conflict of interest.

## Footnotes

1 http://www.fil.ion.ucl.ac.uk/spm

2 http://afni.nimh.nih.gov/pub/dist/doc/manual/AlphaSim.pdf
