# Supplementary material for: Syllogisms delivered in an angry voice lead to improved performance and engagement of a different neural system compared to neutral voice
Source: Front Hum Neurosci. 2015 May 12;9:273. doi: 10.3389/fnhum.2015.00273 (PMC4428274; doi:10.3389/fnhum.2015.00273)
Supplement: Supplementary file 1 [file Data_Sheet_1.DOC]

*Supplementary Material*

**Syllogisms delivered in an angry voice lead to improved performance and engagement of a different neural system compared to neutral voice**

Kathleen W. Smith^1^, Laura-Lee Balkwill^2^, Oshin Vartanian^3^, and Vinod Goel^1, 4^ **^*^**

^1^York University, Toronto, ON, Canada

^2^Humanist Canada, Ottawa, ON, Canada

^3^University of Toronto, Scarborough, ON, Canada

^4^IRCCS Fondazione Ospedale San Camillo, Venice, Italy

Address for correspondence:

Dr. Vinod Goel

Department of Psychology,

Faculty of Health,

York University,

4700 Keele Street,

Toronto, Canada,

M3J 1P3

[vgoel@yorku.ca](mailto:vgoel@yorku.ca)

**Baseline trials.**

**1.1.** We thank a reviewer for raising the issue that because baseline trials are infrequent in the design and because the concluding sentence of such trials is not expected from the premises, the baseline trials may invoke neural processing related to surprise. To respond to this concern, we first address the issue of frequency. In our design, the frequency of baseline trials is 33% of all trials. In a selection of experiments specifically studying surprise, the rate of surprising events has been as follows: 30% of expected targets were omitted (Langner, Kellermann, Boers, Sturm, Willmes, & Eickhoff, 2011), 25% of outcomes did not match expected outcomes (Fletcher et al., 2001), 12.57% of stimuli were oddballs in a novelty-oddball task (Wessel, Danielmeier, Morton, & Ullsperger, 2012), and 10% of face images were presented inverted rather than upright (Egner, Monti, & Summerfield, 2010). Thus, on the basis of frequency of presentation, it is plausible to suggest that participants in our study may have experienced surprise when a baseline was presented instead of a conclusion related to the argument.

The purpose of having baseline trials in our design is to provide a control condition for the analysis of neuroimaging data related to the reasoning time window. The onset of that time window is the end of the concluding sentence (when the speaker has stopped speaking); the duration is from that moment until the individual participants' motor response within each trial. If baseline trials incurred a reaction of surprise, that reaction would arguably have occurred during the hearing of the baseline, when the participant was encountering subject matter unrelated to the premises. Therefore, the neuroimaging analysis would not be affected by an element of surprise in the baseline trials.

**Reasoning time window.**

**2.1. Behavioral results.** The reasoning syllogism count was as follows: Sad: congruent valid 6, congruent invalid 4; Angry: congruent valid 4, congruent invalid 6; Neutral: congruent valid 7, congruent invalid 9, neutral incongruent valid 12, incongruent invalid 12.

**2.2. Neuroimaging results: simple effects.** The contrast (Sad Reasoning – Sad Baseline) yielded relative activation in right caudate nucleus extending into left caudate nucleus, and relative deactivation in left cuneus. The contrast (Angry Reasoning – Angry Baseline) yielded relative activation in right thalamus (temporal) extending into right caudate nucleus, and in left precentral gyrus extending into left primary somatosensory cortex. The contrast (Neutral Reasoning – Neutral Baseline) yielded relative activation in left caudate nucleus, in left inferior frontal gyrus (triangularis extending into orbitalis), in right lingual gyrus (area 17) extending into right calcarine gyrus (area 17), and in right and left insula lobe. The contrast (Emotional Reasoning - Neutral Reasoning) yielded relative deactivation in right superior temporal gyrus. The reverse contrast (Neutral Reasoning - Emotional Reasoning) yielded no clusters surviving the specified extent. The contrast (Sad Reasoning – Neutral Reasoning) yielded relative deactivation in right superior temporal gyrus, and relative activation in left thalamus (prefrontal) and in left precentral gyrus extending into left inferior frontal gyrus (triangularis: area 45). The reverse contrast (Neutral Reasoning – Sad Reasoning) yielded relative activation in right cuneus (area 18). The contrast (Angry Reasoning – Neutral Reasoning) yielded no clusters surviving the specified extent. The reverse contrast (Neutral Reasoning - Angry Reasoning) yielded no clusters surviving the specified threshold and extent. The contrast (Sad Reasoning – Angry Reasoning) yielded relative activation in left insula lobe extending into left inferior frontal gyrus (orbitalis), in right insula lobe extending into right inferior frontal gyrus (triangularis: area 45) and right middle orbital gyrus, in left inferior frontal gyrus (orbitalis), and in right caudate nucleus, and relative deactivation in right middle temporal gyrus. The reverse contrast (Angry Reasoning - Sad Reasoning) yielded relative activation in left cuneus (area 18) extending into right calcarine gyrus (area 17), in right superior parietal lobe, in right supplementary motor area (area 6), and in left superior parietal lobe (area 7).

**2.3. Neuroimaging results: interactions involving reverse contrasts.**

The reverse interaction contrast, [(Neutral Reasoning – Neutral Baseline) – (Emotional Reasoning – Emotional Baseline)] yielded relative deactivation in right superior temporal gyrus extending into right middle temporal gyrus, relative activation in right cuneus extending into left superior occipital gyrus, as well as relative deactivation in left superior temporal gyrus, and in right middle temporal gyrus.

The reverse contrast, [(Neutral Reasoning – Neutral Baseline) – (Sad Reasoning – Sad Baseline)] yielded relative activation in right cuneus extending into left cuneus.

The reverse contrast, [(Neutral Reasoning – Neutral Baseline) – (Angry Reasoning – Angry Baseline)], yielded relative activation in right superior temporal gyrus extending into right middle temporal gyrus. (see Figure 1, Supplementary Material).


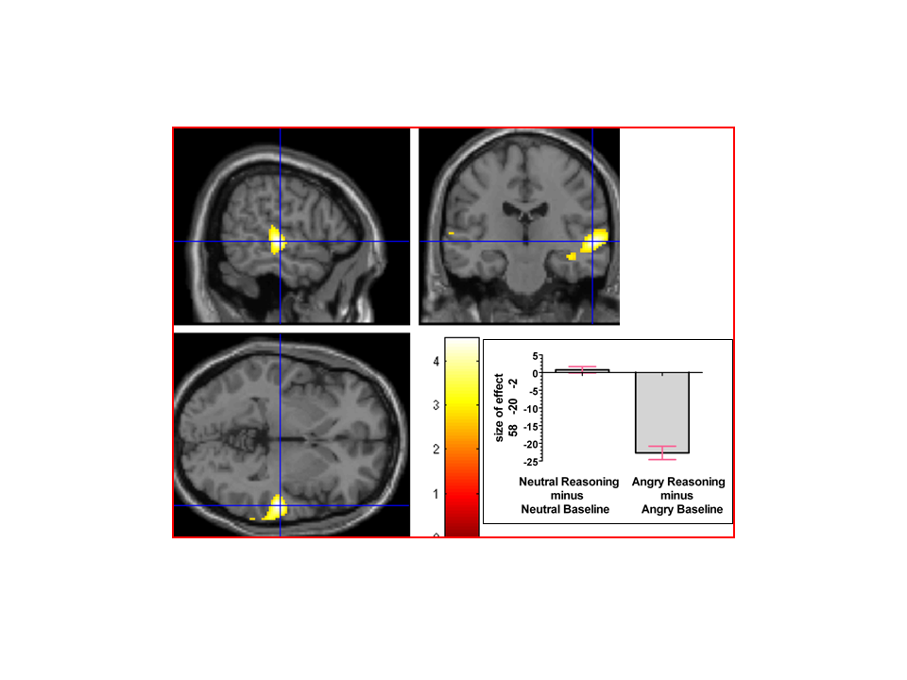


Figure 1, Supplementary Material. The interaction contrast [(Neutral Reasoning – Neutral Baseline) – (Angry Reasoning – Angry Baseline)] elicited activation in right superior temporal gyrus (MNI co-ordinates: 58, -20, -2, cluster size 730 voxels, *Z* = 4.20).

... *continued on next page*

Supplementary Tables

Table 1

Emotion expression (listening) time window: Brain regions identified in the stated comparisons

| **Brain region*^a^*** | |  | | | | | | | | | | | **MNI**  **co-ordinates** | | | | | | |
| --- | --- | --- | --- | --- | --- | --- | --- | --- | --- | --- | --- | --- | --- | --- | --- | --- | --- | --- | --- |
|  | | **k*^b^*** | | ***p^c^*** | | | | | ***Z*** | | | | **x** | | | **y** | | **z** | |
| **Contrast** | | | | | | | | | | | | | | | | | | | |
|  | | | | | | | | | | | | | | | | | | | |
| *Emotion – Neutral* | | | | | | | | | | | | | | | | | | | |
| left hippocampus | | 1315 | | .005 | | 4.93 | | | | | | -30 | | -30 | | | | -10 | |
| left insula lobe | |  | |  | | 4.47 | | | | | | -42 | | -6 | | | | -6 | |
| right posterior insula (ld1) | | 299 | | .343 | | 3.51 | | | | | | 44 | | -2 | | | | -18 | |
| right inferior temporal gyrus | |  | |  | | 3.25 | | | | | | 44 | | 4 | | | | -36 | |
|  | |  | |  | |  | | | | | |  | |  | | | |  | |
| *Neutral – Emotion* | | | | | | | | | | | | | | | | | | | |
| left inferior frontal gyrus (opercularis) | | 408 | | .380 | | 3.48 | | | | | | -42 | | 20 | | | | 32 | |
| left inferior frontal gyrus (triangularis: area 45) | |  | |  | | 3.08 | | | | | | -48 | | 26 | | | | 26 | |
| left precentral gyrus | | 329 | | .380 | | 3.49 | | | | | | -28 | | -4 | | | | 56 | |
| left superior frontal gyrus | |  | |  | | 2.93 | | | | | | -22 | | 0 | | | | 48 | |
|  | |  | |  | | | | | |  |  | | | | |  |  | |  |
| *Sad – Neutral, masked inclusively with Emotion-Neutral at p = .05* | | | | | | | | | | | | | | | | | | |  |
| left hippocampus | | 6766 | | .001 | | | | | | 5.83 | -30 | | | | | -30 | -12 | |  |
| left precuneus | |  | |  | | | | | | 4.61 | -14 | | | | | -54 | 26 | |  |
| right hippocampus | | 1135 | | .110 | | | | | | 4.51 | 40 | | | | | -8 | -24 | |  |
| right inferior temporal gyrus | |  | |  | | | | | | 4.32 | 44 | | | | | 4 | -38 | |  |
| right fusiform gyrus | |  | |  | | | | | | 4.03 | 44 | | | | | -36 | -18 | |  |
| left inferior temporal gyrus | | 207 | | .381 | | | | | | 4.00 | -36 | | | | | -4 | -36 | |  |
| left hippocampus | |  | |  | | | | | | 3.59 | -28 | | | | | -2 | -38 | |  |
| left fusiform gyrus | |  | |  | | | | | | 3.23 | -32 | | | | | -10 | -32 | |  |
| right primary somatosensory cortex (area 3a) | | 187 | | .508 | | | | | | 3.72 | 16 | | | | | -34 | 58 | |  |
| right precentral gyrus (area 6) | |  | |  | | | | | | 2.99 | 28 | | | | | -24 | 58 | |  |
|  |  | | |  | | | |  | | |  | | | | |  |  | |  |
| *Neutral – Sad* | | | | | | | | | | | | | | | | | | |  |
| left superior temporal gyrus (TE 1) | 2612 | | | .001 | | | | 6.71 | | | -50 | | | | | -22 | 6 | |  |
| left middle temporal gyrus |  | |  | | | | 5.50 | | | | -60 | | | | | -14 | -6 | |  |
| right superior temporal gyrus | 2120 | | .001 | | | | 5.94 | | | | 56 | | | | | -10 | -2 | |  |
| right superior temporal gyrus (TE 3) |  | |  | | | | 5.69 | | | | 68 | | | | | -16 | 4 | |  |
| right superior temporal gyrus (TE 1) |  | |  | | | | 4.52 | | | | 48 | | | | | -18 | 4 | |  |
| left cerebellum | 1127 | | .003 | | | | 4.19 | | | | -24 | | | | | -84 | -24 | |  |
| left cerebellum |  | |  | | | | 3.82 | | | | -46 | | | | | -64 | -28 | |  |
| right cerebellar vermis |  | |  | | | | 3.60 | | | | 6 | | | | | -82 | -24 | |  |
| left inferior frontal gyrus (opercularis: area 44) | 499 | | .053 | | | | 3.72 | | | | -48 | | | | | 14 | 32 | |  |
| left calcarine gyrus (area 17) | 404 | | .073 | | | | 3.70 | | | | -2 | | | | | -90 | -4 | |  |
| left calcarine gyrus (area 17) |  | |  | | | | 3.43 | | | | -4 | | | | | -84 | 4 | |  |
| SI Table 1, *continued* | | | | | | | | | | | | | | | | | | |  |
|  |  | |  | | | |  | | | |  | | | | |  |  | |  |
| *Neutral – Sad, continued* | | | | | | | | | | | | | | | | | | |  |
| right cerebellum | 267 | | .147 | | | | 4.06 | | | | 34 | | | | | -60 | -28 | |  |
|  |  | |  | | | |  | | | |  | | | | |  |  | |  |
| *Angry – Neutral, masked inclusively with Emotion-Neutral at p = .05* | | | | | | | | | | | | | | | | | | |  |
| left superior temporal gyrus (TE 1) | 746 | | .145 | | | | 5.01 | | | | -46 | | | | | -14 | 4 | |  |
| left superior temporal gyrus |  | |  | | | | 4.90 | | | | -50 | | | | | -10 | -2 | |  |
| left superior temporal gyrus (TE 1) |  | |  | | | | 4.04 | | | | -38 | | | | | -26 | 4 | |  |
| right superior temporal gyrus | 463 | | .145 | | | | 6.20 | | | | 50 | | | | | -10 | -4 | |  |
| right supramarginal gyrus | 196 | | .657 | | | | 4.35 | | | | 64 | | | | | -28 | 12 | |  |
| right superior temporal gyrus |  | |  | | | | 3.58 | | | | 60 | | | | | -36 | 10 | |  |
|  |  | |  | | | |  | | | |  | | | | |  |  | |  |
| *Neutral - Angry* | | | | | | | | | | | | | | | | | | |  |
| left superior frontal gyrus (area 6) | 1522 | | .005 | | 4.98 | | | | | | -22 | | | | | -10 | 62 | |  |
| left supramarginal gyrus | 332 | | .471 | | 3.70 | | | | | | -52 | | | | | -34 | 48 | |  |
| left supramarginal gyrus |  | |  | | 3.31 | | | | | | -54 | | | | | -52 | 42 | |  |
| right angular gyrus | 197 | | .471 | | 3.70 | | | | | | 48 | | | | | -80 | 24 | |  |
|  |  | |  | |  | | | | | | | |  | |  | | |  | |
| *Sad – Angry, masked inclusively with Emotion-Neutral at p = .05* | | | | | | | | | | | | | | | | | | | |
| left hippocampus | 4155 | | .138 | | 5.24 | | | | | | | | -32 | | -30 | | | -16 | |
| left cuneus |  | |  | | 5.00 | | | | | | | | -8 | | -62 | | | 20 | |
| right hippocampus | 419 | | .142 | | 4.23 | | | | | | | | 36 | | -10 | | | -24 | |
| right inferior temporal gyrus |  | |  | | 3.71 | | | | | | | | 42 | | 6 | | | -40 | |
| right inferior temporal gyrus |  | |  | | 3.28 | | | | | | | | 48 | | -4 | | | -36 | |
|  |  | |  | |  | | | | | | | |  | |  | | |  | |
| *Angry - Sad, masked inclusively with Emotion-Neutral at p = .05* | | | | | | | | | | | | | | | | | | | |
| left superior temporal gyrus (TE 1) | 314 | | .325 | | 5.81 | | | | | | | | -48 | | -12 | | | 2 | |
| left superior temporal gyrus (TE 1) |  | |  | | 4.48 | | | | | | | | -40 | | -26 | | | 2 | |
| left secondary somatosensory cortex (OP4) |  | |  | | 4.16 | | | | | | | | -54 | | -8 | | | 8 | |
| right superior temporal gyrus | 280 | | .325 | | 7.22 | | | | | | | | 50 | | -10 | | | -4 | |
|  |  | |  | |  | | | | | | | |  | |  | | |  | |

*^a^*Brain regions have been identified using SPM Anatomy Toolbox (Eickhoff et al., 2005)

*^b^*All reported clusters (*k* indicates cluster size) are significant at p < .005 for 180 contiguous voxels uncorrected.

*^c^*We also provide the cluster *p*-value, corrected for multiple comparisons using the false discovery rate (FDR; Genovese et al., 2002).

Following the identification of each (significant) cluster, other voxels of activation within that cluster are listed where applicable.

..... *continued on next page*

Table 2

Reasoning time window: Brain regions identified in the stated comparisons

| **Brain region*^a^*** | |  | | | | | | | | | | | **MNI**  **co-ordinates** | | | | | | |
| --- | --- | --- | --- | --- | --- | --- | --- | --- | --- | --- | --- | --- | --- | --- | --- | --- | --- | --- | --- |
|  | | **k*^b^*** | | ***p^c^*** | | | | | ***Z*** | | | | **x** | | | **y** | | **z** | |
| **Contrast** | | | | | | | | | | | | | | | | | | | |
|  | | | | | | | | | | | | | | | | | | | |
| *Reasoning - Baseline* | | | | | | | | | | | | | | | | | | | |
| right insula lobe | | 2658 | | .001 | | 4.60 | | | | | | 32 | | 24 | | | | -2 | |
| right caudate nucleus | |  | |  | | 3.73 | | | | | | 8 | | 14 | | | | 6 | |
| left precentral gyrus | | 444 | | .257 | | 3.74 | | | | | | -32 | | -2 | | | | -46 | |
| left precentral gyrus | |  | |  | | 3.48 | | | | | | -42 | | -2 | | | | 36 | |
| left primary somatosensory cortex (area 3b) | |  | |  | | 3.17 | | | | | | -40 | | -22 | | | | 42 | |
| left insula lobe | | 241 | | .629 | | 3.68 | | | | | | -34 | | 20 | | | | 0 | |
| left inferior frontal gyrus (triangularis) | |  | |  | | 2.94 | | | | | | -48 | | 22 | | | | 2 | |
|  | |  | |  | |  | | | | | |  | |  | | | |  | |
| *Emotional Reasoning – Emotional Baseline* | | | | | | | | | | | | | | | | | | | |
| right thalamus (temporal) | | 2190 | | .001 | | 4.61 | | | | | | 4 | | -2 | | | | 6 | |
| right insula lobe | |  | |  | | 3.79 | | | | | | 32 | | 24 | | | | -2 | |
| left precentral gyrus | | 482 | | .190 | | 4.18 | | | | | | -32 | | -4 | | | | 46 | |
| left precentral gyrus | |  | |  | | 3.27 | | | | | | -42 | | 0 | | | | 36 | |
| left primary somatosensory cortex (area 3b) | |  | |  | | | | | | 3.06 | -42 | | | | | -22 | 42 | |  |
| right middle cingulate cortex | | 223 | | .659 | | | | | | 2.97 | 10 | | | | | 6 | 38 | |  |
|  | |  | |  | | | | | |  |  | | | | |  |  | |  |
| *Sad Reasoning – Sad Baseline* | | | | | | | | | | | | | | | | | | |  |
| right caudate nucleus | | 967 | | .029 | | | | | | 3.95 | 18 | | | | | 24 | 4 | |  |
| left caudate nucleus | |  | |  | | | | | | 3.16 | -12 | | | | | 22 | 8 | |  |
| left cuneus | | 256 | | .679 | | | | | | 3.29 | -18 | | | | | -56 | 26 | |  |
|  | |  | |  | | | | | |  |  | | | | |  |  | |  |
| *Angry Reasoning – Angry Baseline* | | | | | | | | | | | | | | | | | | |  |
| right thalamus (temporal) | | 1326 | | .007 | | | | | | 4.23 | 8 | | | | | -4 | 6 | |  |
| right caudate nucleus | |  | |  | | | | | | 3.26 | 8 | | | | | 14 | 6 | |  |
| left precentral gyrus | | 593 | | .104 | | | | | | 4.21 | -34 | | | | | -4 | 44 | |  |
| left primary somatosensory cortex (area 3b) | |  | |  | | | | 3.41 | | | -42 | | | | | -22 | 40 | |  |
|  |  | | |  | | | |  | | |  | | | | |  |  | |  |
| *Neutral Reasoning – Neutral Baseline* | | | | | | | | | | | | | | | | | | |  |
| left caudate nucleus | 705 | | | .066 | | | | 3.63 | | | -6 | | | | | 10 | 8 | |  |
| left caudate nucleus |  | |  | | | | 3.58 | | | | -10 | | | | | 6 | 16 | |  |
| left inferior frontal gyrus (triangularis) | 297 | | .175 | | | | 3.19 | | | | -52 | | | | | 38 | 0 | |  |
| left inferior frontal gyrus (orbitalis) |  | |  | | | | 3.17 | | | | -48 | | | | | 46 | -8 | |  |
| right lingual gyrus (area 17) | 278 | | .175 | | | | 3.50 | | | | 8 | | | | | -90 | -4 | |  |
| right calcarine gyrus (area 17) |  | |  | | | | 2.71 | | | | 6 | | | | | -78 | 4 | |  |
| right insula lobe | 257 | | .175 | | | | 4.07 | | | | 32 | | | | | 26 | -2 | |  |
| left insula lobe | 179 | | .260 | | | | 3.39 | | | | -36 | | | | | 20 | 2 | |  |
|  |  | |  | | | |  | | | |  | | | | |  |  | |  |
| SI Table 2, *continued* | | | | | | | | | | | | | | | | | | |  |
|  |  | |  | | | |  | | | |  | | | | |  |  | |  |
| *Emotional Reasoning – Neutral Reasoning* | | | | | | | | | | | | | | | | | | |  |
| right superior temporal gyrus (TE 3) | 671 | | .120 | | | | 4.43 | | | | 70 | | | | | -18 | 6 | |  |
| right superior temporal gyrus (TE 3) |  | |  | | | | 2.73 | | | | 58 | | | | | -6 | 10 | |  |
|  |  | |  | | | |  | | | |  | | | | |  |  | |  |
| *Neutral Reasoning – Emotional Reasoning* | | | | | | | | | | | | | | | | | | |  |
| no clusters met extent |  | |  | |  | | | | | |  | | | | |  |  | |  |
|  |  | |  | |  | | | | | |  | | | | |  |  | |  |
| *Sad Reasoning - Neutral Reasoning* | | | | | | | | | | | | | | | | | | |  |
| right superior temporal gyrus (TE 3) | 917 | | .028 | | 3.89 | | | | | | 66 | | | | | -16 | -4 | |  |
| right superior temporal gyrus |  | |  | | 3.88 | | | | | | 66 | | | | | -28 | 0 | |  |
| right superior temporal gyrus (TE 3) |  | |  | | 3.74 | | | | | | 68 | | | | | -16 | 10 | |  |
| left thalamus (prefrontal) | 463 | | .138 | | 3.66 | | | | | | | | -16 | | -4 | | | 12 | |
| left precentral gyrus | 341 | | .193 | | 3.46 | | | | | | | | -50 | | 8 | | | 44 | |
| left inferior frontal gyrus (triangularis: area 45) |  | |  | | 3.17 | | | | | | | | -46 | | 26 | | | 24 | |
|  |  | |  | |  | | | | | | | |  | |  | | |  | |
| *Neutral Reasoning – Sad Reasoning* | | | | | | | | | | | | | | | | | | | |
| right cuneus (area 18) | 556 | | .198 | | 4.49 | | | | | | | | 6 | | -74 | | | 24 | |
|  |  | |  | |  | | | | | | | |  | |  | | |  | |
| *Angry Reasoning – Neutral Reasoning* | | | | | | | | | | | | | | | | | | | |
| no clusters met extent |  | |  | |  | | | | | | | |  | |  | | |  | |
|  |  | |  | |  | | | | | | | |  | |  | | |  | |
| *Neutral Reasoning - Angry Reasoning* | | | | | | | | | | | | | | | | | | | |
| no regions meet threshold or extent |  | |  | |  | | | | | | | |  | |  | | |  | |
|  |  | |  | |  | | | | | | | |  | |  | | |  | |
| *Sad Reasoning – Angry Reasoning* | | | | | | | | | | | | | | | | | | | |
| left insula lobe | 831 | | .049 | | 3.55 | | | | | | | | -34 | | 16 | | | -2 | |
| left inferior frontal gyrus (orbitalis) |  | |  | | 3.51 | | | | | | | | -46 | | 18 | | | -8 | |
| right insula lobe | 625 | | .069 | | 3.31 | | | | | | | | 38 | | 26 | | | -2 | |
| right inferior frontal gyrus (triangularis: area 45) |  | |  | | 3.07 | | | | | | | | 60 | | 28 | | | 2 | |
| right middle orbital gyrus |  | |  | | 3.01 | | | | | | | | 50 | | 46 | | | -8 | |
| left inferior frontal gyrus (orbitalis) | 378 | | .183 | | 3.97 | | | | | | | | -48 | | 42 | | | -6 | |
| right caudate nucleus | 307 | | .216 | | 3.05 | | | | | | | | 16 | | 16 | | | 0 | |
| right middle temporal gyrus | 261 | | .236 | | 3.68 | | | | | | | | 70 | | -32 | | | -6 | |
| right middle temporal gyrus |  | |  | | 3.38 | | | | | | | | 64 | | -32 | | | 0 | |
|  |  | |  | |  | | | | | | | |  | |  | | |  | |
| *Angry Reasoning – Sad Reasoning* | | | | | | | | | | | | | | | | | | | |
| left cuneus (area 18) | 997 | | .024 | | 4.16 | | | | | | | | 4 | | -80 | | | 20 | |
| right calcarine gyrus (area 17) |  | |  | | 3.01 | | | | | | | | 16 | | -78 | | | 2 | |
| right superior parietal lobe | 728 | | .037 | | 3.60 | | | | | | | | 12 | | -52 | | | 62 | |
| right superior parietal lobe |  | |  | | 3.58 | | | | | | | | 8 | | -40 | | | 54 | |
| right supplementary motor area (area 6) | 676 | | .037 | | 4.03 | | | | | | | | 4 | | -14 | | | 66 | |
| left superior parietal lobe (area 7) | 217 | | .427 | | 3.60 | | | | | | | | -10 | | -56 | | | 56 | |
|  |  | |  | |  | | | | | | | |  | |  | | |  | |
|  |  | |  | |  | | | | | | | |  | |  | | |  | |
| SI Table 2, *continued* | | | | | | | | | | | | | | | | | | | |
|  |  | |  | |  | | | | | | | |  | |  | | |  | |
| *(Emotional reasoning – emotional baseline) –*  *(Neutral reasoning – neutral baseline)* | | | | | | | | | | | | | | | | | | | |
| left thalamus (temporal) | 832 | | .055 | | 3.88 | | | | | | | | -8 | | -2 | | | 6 | |
| right thalamus (temporal) |  | |  | | 3.80 | | | | | | | | 8 | | -2 | | | 6 | |
| right caudate nucleus |  | |  | | 3.05 | | | | | | | | 8 | | 12 | | | 6 | |
| right middle cingulate cortex | 311 | | .468 | | 3.47 | | | | | | | | 12 | | 6 | | | 38 | |
| right middle cingulate cortex |  | |  | | 2.70 | | | | | | | | 6 | | 12 | | | 32 | |
|  |  | |  | |  | | | | | | | |  | |  | | |  | |
| *(Neutral reasoning – neutral baseline) - (Emotional reasoning – emotional baseline)* | | | | | | | | | | | | | | | | | | | |
| right superior temporal gyrus | 961 | | .027 | | 4.90 | | | | | | | | 68 | | -28 | | | 2 | |
| right middle temporal gyrus |  | |  | | 3.51 | | | | | | | | 54 | | -24 | | | -16 | |
| right middle temporal gyrus |  | |  | | 3.15 | | | | | | | | 68 | | -52 | | | -6 | |
| right cuneus | 423 | | .210 | | 3.65 | | | | | | | | 14 | | -84 | | | 40 | |
| left superior occipital gyrus |  | |  | | 2.75 | | | | | | | | -4 | | -82 | | | 42 | |
| left superior temporal gyrus | 324 | | .258 | | 3.65 | | | | | | | | -60 | | -30 | | | 8 | |
| right middle temporal gyrus | 179 | | .541 | | 3.42 | | | | | | | | 54 | | -58 | | | 8 | |
|  |  | |  | |  | | | | | | | |  | |  | | |  | |
| *(Sad Reasoning – Sad Baseline) - (Neutral reasoning – neutral baseline)* | | | | | | | | | | | | | | | | | | | |
| no clusters met extent |  | |  | |  | | | | | | | |  | |  | | |  | |
|  |  | |  | |  | | | | | | | |  | |  | | |  | |
| *(Neutral reasoning – neutral baseline) - (Sad Reasoning – Sad Baseline)* | | | | | | | | | | | | | | | | | | | |
| right cuneus | 378 | | .306 | | 3.62 | | | | | | | | 14 | | -84 | | | 42 | |
| left cuneus |  | |  | | 2.66 | | | | | | | | -2 | | -88 | | | 40 | |
|  |  | |  | |  | | | | | | | |  | |  | | |  | |
| *(Angry Reasoning – Angry Baseline) - (Neutral reasoning – neutral baseline)* | | | | | | | | | | | | | | | | | | | |
| right superior frontal gyrus | 611 | | .172 | | 3.43 | | | | | | | | 26 | | 22 | | | 46 | |
| right thalamus (prefrontal) | 220 | | .899 | | 3.67 | | | | | | | | 12 | | -4 | | | 8 | |
|  |  | |  | |  | | | | | | | |  | |  | | |  | |
| *(Neutral reasoning – neutral baseline) - (Angry Reasoning – Angry Baseline)* | | | | | | | | | | | | | | | | | | | |
| right superior temporal gyrus | 730 | | .063 | | 4.20 | | | | | | | | 58 | | -20 | | | -2 | |
| right middle temporal gyrus |  | |  | | 2.98 | | | | | | | | 70 | | -42 | | | 2 | |
|  |  | |  | |  | | | | | | | |  | |  | | |  | |
| *(Sad Reasoning – Sad Baseline) - (Angry Reasoning – Angry Baseline)* | | | | | | | | | | | | | | | | | | | |
| no clusters met extent |  | |  | |  | | | | | | | |  | |  | | |  | |
| *(Angry Reasoning – Angry Baseline) - (Sad Reasoning – Sad Baseline)* | | | | | | | | | | | | | | | | | | | |
| no clusters met extent |  | |  | |  | | | | | | | |  | |  | | |  | |
|  |  | |  | |  | | | | | | | |  | |  | | |  | |
| *Conjunction analysis of (Sad Reasoning – Sad Baseline) - (Neutral reasoning – neutral baseline)*  *and*  *(Angry Reasoning – Angry Baseline) - (Neutral reasoning – neutral baseline)* | | | | | | | | | | | | | | | | | | | |
| no suprathreshold clusters |  | |  | |  | | | | | | | |  | |  | | |  | |
|  |  | |  | |  | | | | | | | |  | |  | | |  | |

*^a^*Brain regions have been identified using SPM Anatomy Toolbox (Eickhoff et al., 2005)

*^b^*All reported clusters (*k* indicates cluster size) are significant at p < .005 for 180 contiguous voxels uncorrected.

*^c^*We also provide the cluster *p*-value, corrected for multiple comparisons using the false discovery rate (FDR; Genovese et al., 2002).

Following the identification of each (significant) cluster, other voxels of activation within that cluster are listed where applicable.

# Appendix A: Procedure for obtaining ratings of 'Tone of Voice' stimuli

**A.1. Method**

**A.1.1. Participants.** Data were acquired from 15 volunteer participants (7 males, 8 females), recruited on a university campus. Education levels ranged from completed high school to completed graduate degrees, with a mean of 15.2 (*SD* = 2.11) years of education. Ages ranged from 18 to 67 (mean 24.4 years, *SD* 12.5 years, median and mode 20 years). No participant reported a hearing problem. All participants gave informed consent. The study was approved by the York University Research Human Participants Ethics Committee.

**A. 1. 2. Stimuli.** Recordings were made by a female speaker (L.-L. B.) of syllogisms with neutral content, in the following tones of voice: Neutral 60, Sad 30, Angry 30, Peaceful 30, Happy 15, and Elated 15.[[1]](#footnote-2) Ten sets of 18 stimuli each were created; thus, all stimuli were represented once.

Guidelines for set creation were as follows:

* At least 1 'reasoning' and 1 'baseline' for each of Sad and Angry in each set.

* At least 2 'reasoning' and 2 'baseline' for Neutral in each set.

* Six sets (#1, 3, 5, 7, 9, 10) should begin with a 'reasoning' stimulus and 4 (#2, 4, 6, 8)

should begin with a 'baseline' stimulus.

*Every third stimulus in each set would be drawn from the Happy Elated Peaceful group

(2 peaceful reasoning, 1 peaceful baseline, 3 of elated and/or happy, with at

least one of each, including 2 'reasoning' and 1 'baseline').

**A.1. 3. Procedure.** Two types of ratings presentations were administered to each participant, in counterbalanced order. Thus, each participant was presented with 2 of the sets.

In one presentation type (see Table A1), participants rated each stimulus by listening to it and then circling one of

Sad Angry Neutral Happy Peaceful Elated

on a worksheet.

After circling one term, the participant indicated a 3, a 2, or a 1 beside it. '3' indicated they were definite about their choice; '2' indicated they felt their choice was good enough, and '1' indicated 'forced choice'. If a '1' was indicated, the participant was asked to write down their preferred term. The order of these six terms was randomised for each trial. Also, the order of presentation of the 'word orders on the worksheets' was randomised across participants.

Table A1: Presentation Type 1

| On each trial, circle one choice.  Then put "1" for maybe, "2" for yes, or "3" for definitely. | | | | | | | | |  | | --- | |
| --- | --- | --- | --- | --- | --- | --- | --- | --- | --- |
| IF you choose "maybe", add a comment to indicate what emotion label would be a better choice. | | | | | | | |  |
|  |  |  |  |  |  |  |  |  |
| ONE |  |  |  |  |  |  |  |  |
|  | Happy | Angry | Neutral | Peaceful | Elated | Sad |  |  |
|  |  |  |  |  |  |  |  |  |
|  |  |  |  |  |  |  |  |  |
| TWO |  |  |  |  |  |  |  |  |
|  | Angry | Happy | Peaceful | Elated | Sad | Neutral |  |  |
|  |  |  |  |  |  |  |  |  |
|  |  |  |  |  |  |  |  |  |
| THREE |  |  |  |  |  |  |  |  |
|  | Angry | Elated | Peaceful | Neutral | Sad | Happy |  |  |
|  |  |  |  |  |  |  |  |  |
|  |  |  |  |  |  |  |  |  |
| FOUR |  |  |  |  |  |  |  |  |
|  | Neutral | Happy | Elated | Angry | Sad | Peaceful |  |  |
|  |  |  |  |  |  |  |  |  |
|  |  |  |  |  |  |  |  |  |
| FIVE |  |  |  |  |  |  |  |  |
|  | Happy | Angry | Sad | Peaceful | Neutral | Elated |  |  |
|  |  |  |  |  |  |  |  |  |
|  |  |  |  |  |  |  |  |  |
| SIX |  |  |  |  |  |  |  |  |
|  | Sad | Angry | Peaceful | Elated | Neutral | Happy |  |  |
|  |  |  |  |  |  |  |  |  |

Note: There were 18 rows altogether.

In the other presentation type (see Table A2), the worksheet indicated

ACTIVE PASSIVE

Negative Neutral Positive Negative Neutral Positive

Participants were instructed to circle one of the six terms, and use the '3', '2', and '1' numbering system. If ‘1’ was indicated, the participant was asked to write down their preferred term.

Each set of 18 stimuli was placed in a separate folder on a Macintosh computer. Folders were named using a numeric code. Within each folder, each stimulus file was identified by a number from 1 to 18.

After the participant signed the consent form, the researcher read the instructions for the first presentation type (counterbalanced order), and trained the participant using six stimuli that were not subsequently presented to that participant for rating. Stimuli were played on a Macintosh computer with volume set to maximum; no headphones were used. It was the researcher who opened the appropriate training folder.

Participants were instructed to focus on the speaker’s tone of voice, as the content would be irrelevant, and to listen to each stimulus file once only.

After training, the researcher closed the training folder and opened the folder containing the appropriate first presentation set for that participant. The researcher then went to an adjoining room, and listened (while consulting detailed notes) as the participant played each stimulus, to ensure that the participant was listening to the stimuli in the correct order. Part two of the experiment proceeded in the same manner; the researcher trained the participant on the second presentation type, using a different set of six stimuli, and the participant proceeded to rate the second presentation type.

Clarification of the terms “active” and “passive” (presentation type 2) was provided to all participants as follows:

“Here is a chart to help me explain the task. While you are listening, first decide whether the tone of voice seems to suggest that the speaker is 'active' or 'passive'.

By 'passive', I mean that this is how someone might sound if they were completely in private, and were just thinking these thoughts rather than saying them out loud. Or, they might be with someone else, but the tone of voice doesn't suggest that they are about to get up and do anything.

By 'active', I mean that the tone of voice suggests that the person is more likely to be standing up and ready to do something, or perhaps is sitting down but is nevertheless actively engaged in doing something. The activity might have nothing to do with what she is actually saying; it's just that she sounds like she is up and ready to do something.

Some sets will sound more 'negative' and some more 'positive'. Also, there will be some that don't seem to lean either way; they are more neutral.

Table A2: Presentation Type 2

| On each trial, circle one choice. Then put "1" for maybe, "2" for yes, or "3" for definitely. | | | | | | | | | |  | | --- | | |
| --- | --- | --- | --- | --- | --- | --- | --- | --- | --- | --- | --- |
| IF you choose "maybe", add a comment to indicate what emotion label would be a better choice. | | | | | | | | | | |
|  |  |  |  |  |  |  |  |  | |  |
| # 1 |  | ACTIVE |  |  |  | PASSIVE |  |  | |  |
|  | Negative | Neutral | Positive |  | Negative | Neutral | Positive |  | |  |
|  |  |  |  |  |  |  |  |  | |  |
|  |  |  |  |  |  |  |  |  | |  |
| # 2 |  | ACTIVE |  |  |  | PASSIVE |  |  | |  |
|  | Negative | Neutral | Positive |  | Negative | Neutral | Positive |  | |  |
|  |  |  |  |  |  |  |  |  | |  |
|  |  |  |  |  |  |  |  |  | |  |
| # 3 |  | ACTIVE |  |  |  | PASSIVE |  |  | |  |
|  | Negative | Neutral | Positive |  | Negative | Neutral | Positive |  | |  |
|  |  |  |  |  |  |  |  |  | |  |
|  |  |  |  |  |  |  |  |  | |  |
| # 4 |  | ACTIVE |  |  |  | PASSIVE |  |  | |  |
|  | Negative | Neutral | Positive |  | Negative | Neutral | Positive |  | |  |
|  |  |  |  |  |  |  |  |  | |  |
|  |  |  |  |  |  |  |  |  | |  |
| # 5 |  | ACTIVE |  |  |  | PASSIVE |  |  | |  |
|  | Negative | Neutral | Positive |  | Negative | Neutral | Positive |  | |  |
|  |  |  |  |  |  |  |  |  | |  |
|  |  |  |  |  |  |  |  |  | |  |
| # 6 |  | ACTIVE |  |  |  | PASSIVE |  |  | |  |
|  | Negative | Neutral | Positive |  | Negative | Neutral | Positive |  | |  |
|  |  |  |  |  |  |  |  |  | |  |

Note: There were 18 rows altogether.

If you hear a set that doesn't lean towards 'negative' or 'positive' how do you rate it as being 'active' or 'passive'? Well, 'active neutral' might for instance sound businesslike, the way the teller at the bank might talk to you when they are telling you about a new product. On the other hand, 'passive neutral' might be very private, but maybe the person is tired, so they are no longer really feeling either very unhappy or very happy, they are just kind of at rest.

I'm showing all these possibilities on a pie chart to suggest that there is a continuum.”

After the participant completed both sets, (s)he completed a short demographics information form and was debriefed. The experiment took approximately 20 minutes per participant.

**A.2. Results**

Sets #1-10 had been presented a total of three times across the experiment; thus, three independent ratings were obtained for each 'Tone of Voice' stimulus.

# Sad

100% agreement (3 out of 3) on 24 of the 30 stimuli.

66.6% agreement (2 out of 3) on 5 of the 30 stimuli.

On each of these, the 'dissenter' chose "Active negative 2".

On one stimulus out of 30,

one person rated it as ‘definitely sad’

one as ‘yes Active negative’

and one as ‘definitely Active negative’.

# Angry

100% agreement (3 out of 3) on 26 of the 30 stimuli.

66.6% agreement (2 out of 3) on 3 of the 30 stimuli.

On one stimulus out of 30, one person rated it as “definitely angry”, one as “annoyed” (written response) and one as “definitely passive”.

# Neutral

100% agreement (3 out of 3) on 51 of the 60 stimuli.

66.6% agreement (2 out of 3) on 9 of the 60 stimuli.

For these, the 3^rd^ rating was:

definitely passive positive: n = 4

yes passive positive: n = 1

yes happy: n = 1

definitely active positive: n = 2

1. The peaceful, happy, and elated stimuli were not used in the experiment, but were included in this ‘ratings’ pilot study to reduce any possible demand characteristics. [↑](#footnote-ref-2)
